# Association between the systemic immune-inflammation index and obesity among adults: Insights from the NHANES 2017–2018

**DOI:** 10.1371/journal.pone.0308288

**Published:** 2024-08-08

**Authors:** Yanmei Yu, Tongcai Tan, Wei Yang, Zhitao Xu, Yong Liu

**Affiliations:** Department of Rehabilitation Medicine, Center for Rehabilitation Medicine, Rehabilitation & Sports Medicine Research Institute of Zhejiang Province, Zhejiang Provincial People’s Hospital (Affiliated People’s Hospital), Hangzhou Medical College, Hangzhou, Zhejiang, China; Federal Medical Centre Umuahia, NIGERIA

## Abstract

**Background:**

Inflammation is an important causative factor of obesity. This study aimed to explore the possible association between the systemic immune-inflammatory index, a novel indicator of inflammation, and obesity.

**Methods:**

Data were collected from 4395 participants of the National Health and Nutrition Examination Survey 2017–2018 aged ≥ 20 years. The systemic immune-inflammatory index was calculated by multiplying the platelet count by the neutrophil-to-lymphocyte ratio. Obesity was defined as a body mass index ≥ 30 kg/m^2^.

**Results:**

A significant positive correlation was observed between the systemic immune-inflammatory index and body mass index following multivariate linear regression analysis (β = 1.75; 95% confidence interval = 1.16–2.33), which was greatest in adults aged < 60 years without hypertension and diabetes. Smoothed curve fitting and threshold effect analysis were used to characterize the nonlinear association between the systemic immune-inflammatory index and body mass index, and the inflection point was found to be 729.3.

**Conclusions:**

The systemic immune-inflammatory index is positively associated with body mass index among adults in the United States and has the potential to enhance efforts to prevent adult obesity.

## Introduction

The prevalence of obesity, as determined by the body mass index (BMI) [1], continues to rise, and it currently affects 39.5% of the population of the United States [2]. Obesity can be caused by various factors and is associated with several health issues, including type 2 diabetes, asthma, cardiovascular disease, chronic obstructive pulmonary disease, musculoskeletal disorders, and cancer [3]. Obesity is linked to a 29% increase in deaths from all causes [4]. Consequently, managing and preventing obesity have become critical public health challenges. The systemic immune inflammation Index (SII) is a novel indicator of inflammation proposed by Hu et al. [5]. It combines peripheral blood neutrophil, lymphocyte, and platelet counts [6] to reflect the inflammatory and immunological status of the body [7]. Numerous studies have identified the SII as an important prognostic biomarker for various cancers, including hepatocellular carcinoma [8], gastric carcinoma [9], pancreatic carcinoma [10], non-small cell lung carcinoma [11], bladder carcinoma [12], and medullary thyroid carcinoma [13]. In addition, the SII has strong diagnostic and prognostic values for diseases associated with immune dysfunction-induced inflammation [14,15]. Higher SII scores indicate a constantly activated immune system and therefore an increased risk of chronic inflammation [16], which can impair glucose metabolism and metabolic balance [17]. Inflammatory mediators are often detected in the adipose tissue of obese individuals, and chronic low-level inflammation is the primary feature of obesity [18]. Some studies have detected higher plasma concentrations of inflammatory mediators, including C-reactive protein (CRP), interleukin-6 (IL-6), and tumor necrosis factor-α (TNF-α), in obese individuals [19]. Inflammation is therefore considered a major pathogenic component of obesity [20]; however, the association between the SII and obesity remains unclear.

We conducted a cross-sectional study using data from the National Health and Nutrition Examination Survey (NHANES) 2017–2018, which aimed to investigate the association between the SII and BMI and ascertain the ability of the SII to predict obesity.

## Materials and methods

### Study population

**Fig 1:**
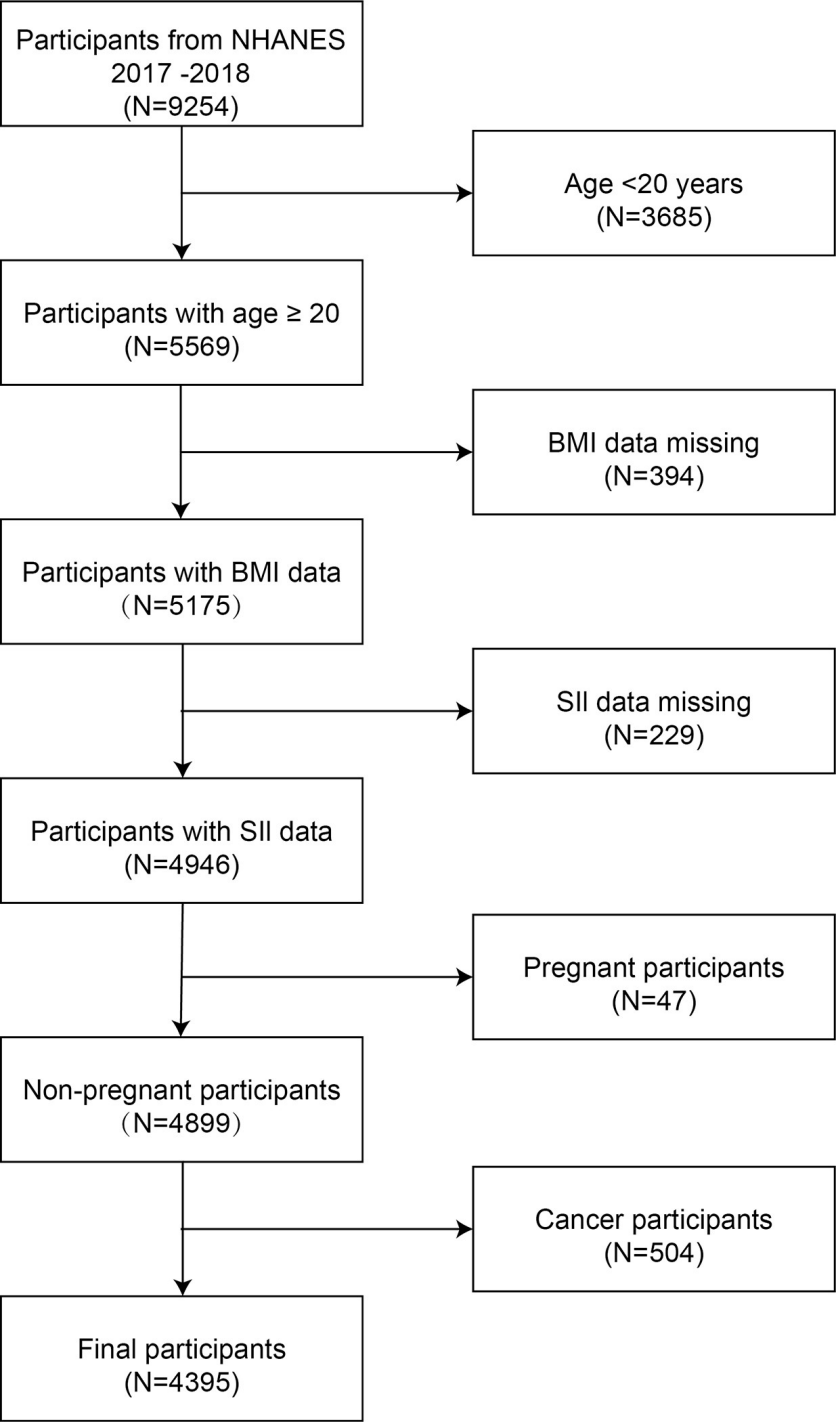
The flowchart of participant selection.NHANES. National Health and Nutrition Examination Survey; BMI, body mass index; SII, systemic immune-inflammation index.

NHANES (https://www.cdc.gov/nchs/nhanes/index.htm) is a comprehensive, ongoing study conducted in the United States by the Centers for Disease Control and Prevention. The NHANES collects data on health and nutrition through detailed household interviews, physical examinations, and laboratory tests, using a multistage probability sampling method to ensure a nationally representative sample. Strict quality control during data collection and processing ensures the accuracy and reliability of the data, which are collected by trained healthcare professionals [21]. The NHANES was reviewed and approved by the Board of Institutional Review at the National Center for Health Statistics and each participant provided written informed consent. The present study used data from the 2017 to 2018 cycle of the NHANES. We excluded 3685 participants aged < 20 years, 394 participants without BMI data, 229 participants without SII data, 47 pregnant participants, and 504 participants with a history of cancer. Finally, 4395 participants were included in the analysis. The workflow for sample selection is presented in Fig 1.

### SII

The SII was calculated by counting whole blood cells using a fully automated DxH 800 Hematology Analyzer (Beckman Coulter, Brea, CA, USA), full details of this protocol can be found on the NHANES website [22]. The SII was calculated using the following formula: SII = neutrophil count × platelet cell count /lymphocyte count.

### BMI measurement and obesity diagnosis

BMI was calculated using height and weight measurements obtained from the physical examination component of the NHANES, as weight in kilograms divided by the square of height in meters (kg/m^2^) [23]. Participants were categorized according to World Health Organization guidelines: normal weight, BMI of 18.5–24.9 kg/m^2^; overweight, BMI ≥ 25 kg/m^2^; and obese, BMI ≥ 30 kg/m^2^.

### Covariables

Covariates that may affect the association between the SII and BMI, including age, race, sex, education level, poverty-income ratio, marital status, smoked at least 100 cigarettes in life, alcohol use, physical activity, hypertension, diabetes, waist circumference, HDL (high-density lipoprotein), triglycerides, LDL (low-density lipoprotein), total cholesterol, and high-sensitivity CRP, were also included in our study. Among the participants, race was classified as Non-Hispanic White, Non-Hispanic Black, Mexican American, and other races. Education levels were classified into three categories: less than high school, high school, and more than high school. The income-to-poverty ratio was calculated using poverty guidelines adjusted for family size and then categorized into three distinct groups to represent participants’ socioeconomic status: below 1.5, between 1.5 and 3.5, and above 3.5 [24]. Marital status was categorized into three groups: married/living with a partner, widowed/divorced/separated, and never married. Participants who smoked more than 100 cigarettes in their lifetime were classified as smokers; others were considered non-smokers [25]. Alcohol use was evaluated through a questionnaire where participants self-reported their average drink intake on days they consumed alcohol over the past year. Physical activity is defined as whether the work involves moderate-intensity activities that result in small increases in breathing or heart rate [26]. Diabetes mellitus is diagnosed based on the presence of any one of the following criteria: (1) a physician’s diagnosis of diabetes mellitus; (2) current usage of hypoglycemic medication; (3) a glycosylated hemoglobin (HbA1c) level of ≥6.5%; (4) a fasting blood glucose level of ≥7.0 mmol/L [27]. A diagnosis of hypertension can be established through any of the following criteria: (1) a physician’s diagnosis of hypertension; (2) current use of prescription medication for hypertension; (3) a systolic blood pressure of ≥ 140 mmHg and/or a diastolic blood pressure of ≥ 90 mmHg, as measured in the participant [27]. Waist circumference was measured using a tape measure positioned at the uppermost lateral border of the iliac crest. HDL, triglycerides, and total cholesterol were measured enzymatically [28]. LDL was calculated using the Friedewald equation: LDL = TC—HDL—TG/5 [29]. High-sensitivity CRP was measured by immunoturbidimetric assay.

### Statistical analysis

Categorical characteristics are shown as percentages, while continuous variables are expressed as means (standard deviation). Weighted multivariate linear analysis was performed to investigate correlations between the SII and BMI. The trend of the linear correlation between the SII and BMI was investigated with a trend test after the conversion of the SII score from continuous to categorical variables using quartiles. The association between the SII and BMI in participants of varying sex, age, race, education level, poverty-income ratio, smoking status, hypertension, and diabetes was examined using subgroup analysis, and the consistency of the associations was examined using interaction tests. Smoothed curve fitting was used to investigate the nonlinear relationship between the SII and BMI. To investigate threshold effects, a two-piecewise linear regression model was applied. Statistical analyses were performed using R software version 4.2 and EmpowerStats software version 5.0. Statistical significance was set at *p* < 0.05.

## Results

### Baseline characteristics

Table 1 summarizes the weighted baseline characteristics of the 4395 participants grouped by SII quartiles. The participants had an average age of 49.93 (17.16) years, with 51.31% being female and 32.2% identifying as non-Hispanic white. Participants had a mean SII of 502.51 (314.25) and were categorized into the following quartiles: 1, < 308.76; 2, 308.76–437.19; 3, 437.19–613.13; and 4, > 613.13.

**Table 1:** Characteristics of participants in the NHANES 2017–2018 cycle.

Characteristics	Systemic immune-inflammation index				*p*-value
	Q1	Q2	Q3	Q4	
Participants	N = 1099	N = 1098	N = 1099	N = 1099	
Age (years)	46.47 ± 16.45	45.65 ± 16.48	47.13 ± 16.86	47.47 ± 16.68	0.0446
Sex, (%)					<0.0001
Male	56.78	54.69	45.21	41.52	
Female	43.22	45.31	54.79	58.48	
Race/ethnicity, (%)					<0.0001
Non-Hispanic White	50.35	60.09	63.39	66.42	
Non-Hispanic Black	20.75	10.66	8.66	8.18	
Mexican American	8.26	11.02	9.08	8.94	
Other races	20.64	18.24	18.87	16.46	
Education level, (%)					0.3565
< high school	10.97	12.19	11.54	10.88	
High school	26.98	25.51	28.73	29.72	
> high school	62.06	62.30	59.73	59.40	
Income to poverty ratio, (%)					0.0785
<1.5	22.65	20.63	20.24	24.15	
1.5–3.5	38.16	38.11	40.04	40.18	
>3.5	39.19	41.26	39.72	35.67	
Marital status, (%)					0.4142
Married/Living with partner	62.07	62.42	64.45	61.07	
Widowed/Divorced/Separated	16.43	16.67	17.08	18.80	
Never married	21.50	20.90	18.47	20.13	
Smoked at least 100 cigarettes, (%)					0.0012
Yes	41.12	37.70	42.08	45.82	
No	58.88	62.30	57.92	54.18	
Physical activity, (%)					0.8967
Yes	48.90	49.09	48.98	50.31	
No	51.10	50.91	51.02	49.69	
Hypertension, (%)					<0.0001
Yes	35.20	34.25	35.71	45.74	
No	64.80	65.75	64.29	54.26	
Diabetes, (%)					0.0002
Yes	12.52	14.78	15.61	19.36	
No	87.48	85.22	84.39	80.64	
Alcohol use	2.11 ± 1.89	2.19 ± 1.93	2.10 ± 1.87	2.30 ± 2.26	0.0740
HDL (mg/dL)	54.66 ± 16.47	53.30 ± 14.66	52.52 ± 14.80	53.08 ± 15.02	0.0134
Triglyceride (mg/ dL)	101.46 ±100.35	103.50 ± 72.67	101.47 ± 51.33	100.16 ± 44.11	0.7052
LDL (mg/dL)	109.21 ± 23.43	111.32 ± 24.84	108.41 ± 25.43	108.50 ± 22.72	0.0124
Total cholesterol (mg/dL)	187.32 ± 37.71	192.08 ± 40.24	187.31 ± 40.03	187.46 ± 37.52	0.0062
Waist circumference, cm	96.93 ± 15.36	99.38 ± 16.52	100.96 ± 17.32	104.45 ± 18.29	<0.0001
High-Sensitivity CRP	2.24±3.03	2.60±3.72	3.48±4.96	6.46±12.16	<0.0001
BMI, kg/m^2^					<0.0001
<25	32.19	26.87	25.19	22.41	
25–30	34.35	33.93	30.62	25.58	
>30	33.45	39.20	44.18	52.01	

Mean ± SD for continuous variables: The p-value was calculated by the weighted linear regression model. (%) for categorical variables: The P value was calculated by the weighted chi-square test. Abbreviation: HDL,high-density lipoprotein; LDL, Low-density lipoprotein; BMI, body mass index.

Statistically significant variations were observed among the SII quartiles with respect to age, sex, race, smoking, hypertension, diabetes, HDL, LDL, total cholesterol, waist circumference, high-sensitivity CRP, and BMI (*p* < 0.05). Individuals in quartile 4 were more likely to be older, female, non-Hispanic white, have smoked less than 100 cigarettes, be free of diabetes and hypertension, have a larger waist circumference, have higher levels of high-sensitivity CRP, and have a BMI ≥ 30 kg/m^2^ (*p* < 0.05).

### Association between the SII and BMI

**Fig 2:**
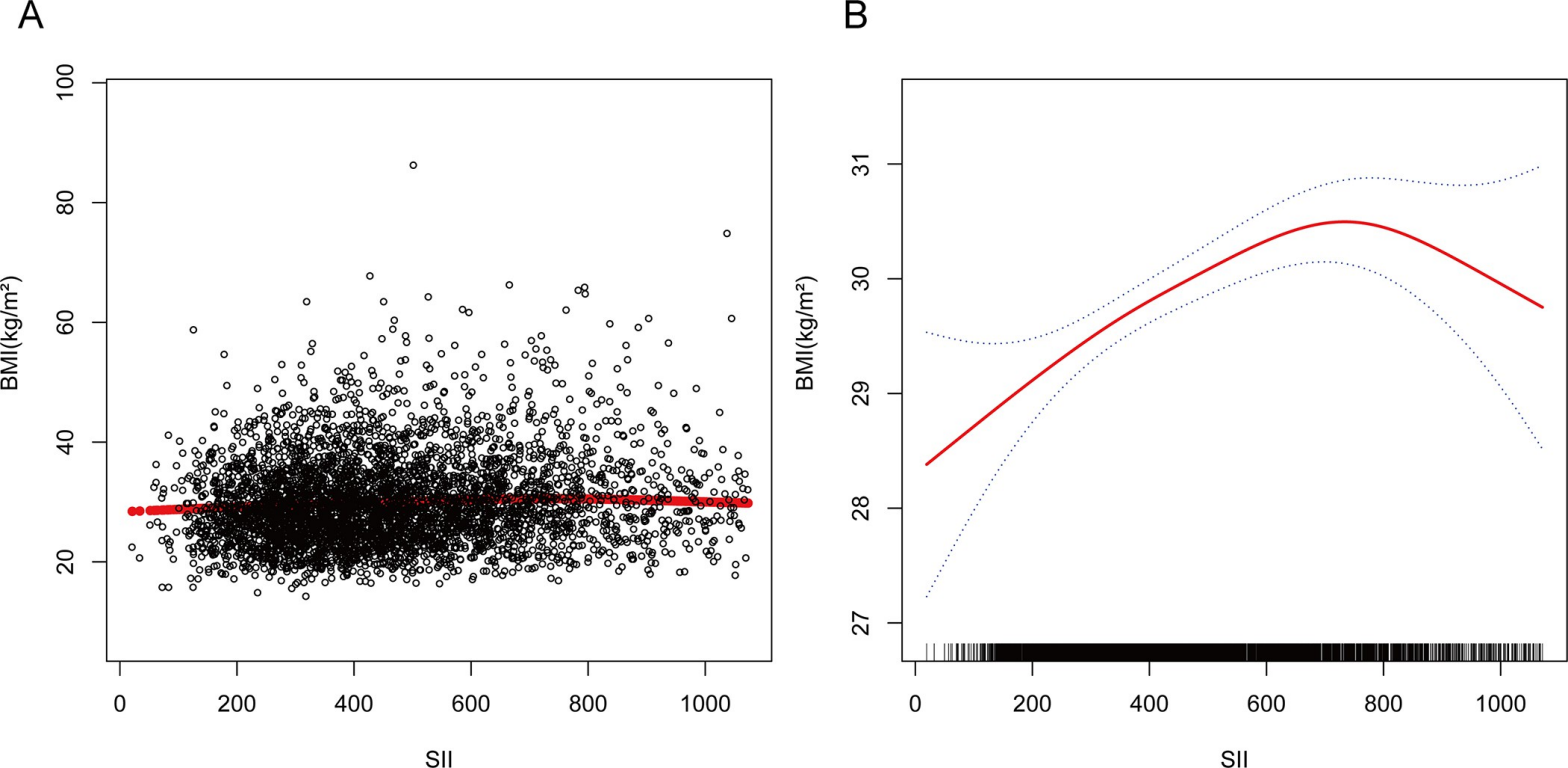
The nonlinear associations between SII and BMI. (A) Each black point represents a sample. (B)The solid red line represents the smooth curve fit between variables. Blue bands represent the 95% confidence interval from the fit.

The study utilized multivariate linear regression analysis to explore the potential autonomous correlation between the SII and BMI; the results of this analysis are presented in Table 2. The SII was scaled by dividing it by 100, which amplified the effect size 100 times. Significant correlations between obesity and higher SII scores were observed in model 1 (β = 0.22; 95% confidence interval [CI] = 0.15–0.29, *p* < 0.001), model 2 (β = 0.23; 95% CI = 0.16–0.30, *p* < 0.001), and the fully adjusted model (β = 0.06; 95% CI = 0.00–0.12, *p* < 0.05). A 0.06 kg/m^2^ rise in BMI was positively correlated with a 100-unit increase in SII. After categorization of the SII, quartiles 2, 3, and 4 showed increases of 0.61 kg/m^2^ (95% CI = 0.04–1.18, *p* < 0.05), 1.29 kg/m^2^ (95% CI = 0.72–1.86, *p* < 0.001), and 1.75 kg/m^2^ (95% CI = 1.16–2.33, *p* < 0.001), respectively, compared with quartile 1. In all three models, this trend was statistically significant (*p* < 0.001). Furthermore, an inverted U-shaped association between the SII and BMI was revealed by smoothed curve fitting (Fig 2), with 729.3 serving as the inflection point (Table 3).

**Table 2:** Association between the systemic immune-inflammation index and BMI.

	Crude	Partially	Fully
	Model	Adjusted Model	Adjusted Model
	(Model 1)	(Model 2)	(Model 3)
	β(95%CI), *p*-value	β(95% CI), *p*-value	β(95% CI), *p*-value
SII/100	0.22 (0.15, 0.29)	0.23 (0.16, 0.30)	0.06 (0.00, 0.12)
SII quartile			
Quartile 1	Reference	Reference	Reference
Quartile 2	0.93 (0.30, 1.56)	1.08 (0.45, 1.71)	0.61 (0.04, 1.18)
Quartile 3	1.88 (1.25, 2.51)	2.09 (1.46, 2.73)	1.29 (0.72, 1.86)
Quartile 4	3.20 (2.57, 3.82)	3.39 (2.76, 4.03)	1.75 (1.16, 2.33)
*p* for trend	<0.001	<0.001	<0.001

Model 1: No covariates were adjusted.

Model 2: Age, sex, and race were adjusted.

Model 3: Age, sex, race, education level, income-to-poverty ratio, marital status, smoking status, alcohol use, physical activity, hypertension, diabetes, HDL, triglyceride, total cholesterol, and High-Sensitivity CRP were adjusted.

Abbreviations: SII, systemic immune-inflammation index; BMI, body mass index; HDL,high-density lipoprotein; LDL, low-density lipoprotein.

**Table 3:** Threshold effect analysis of SII on BMI using the two-piecewise linear regression model.

BMI (kg/m^2^)	Adjusted β (95% CI) *p*-value
SII	
Inflection point	729.3
SII<729.3	0.00 (0.00, 0.01) <0.0001
SII>729.3	-0.01 (-0.01, -0.00) <0.0001
Log likelihood ratio	<0.001

Age, sex, race, educational level, marital status, poverty-income ratio, smoking status, alcohol use, physical activity, hypertension, diabetes, HDL, triglyceride, total cholesterol, and High-Sensitivity CRP were adjusted. BMI, body mass index; SII, systemic immune-inflammation index.

### Subgroup analyses

**Fig 3:**
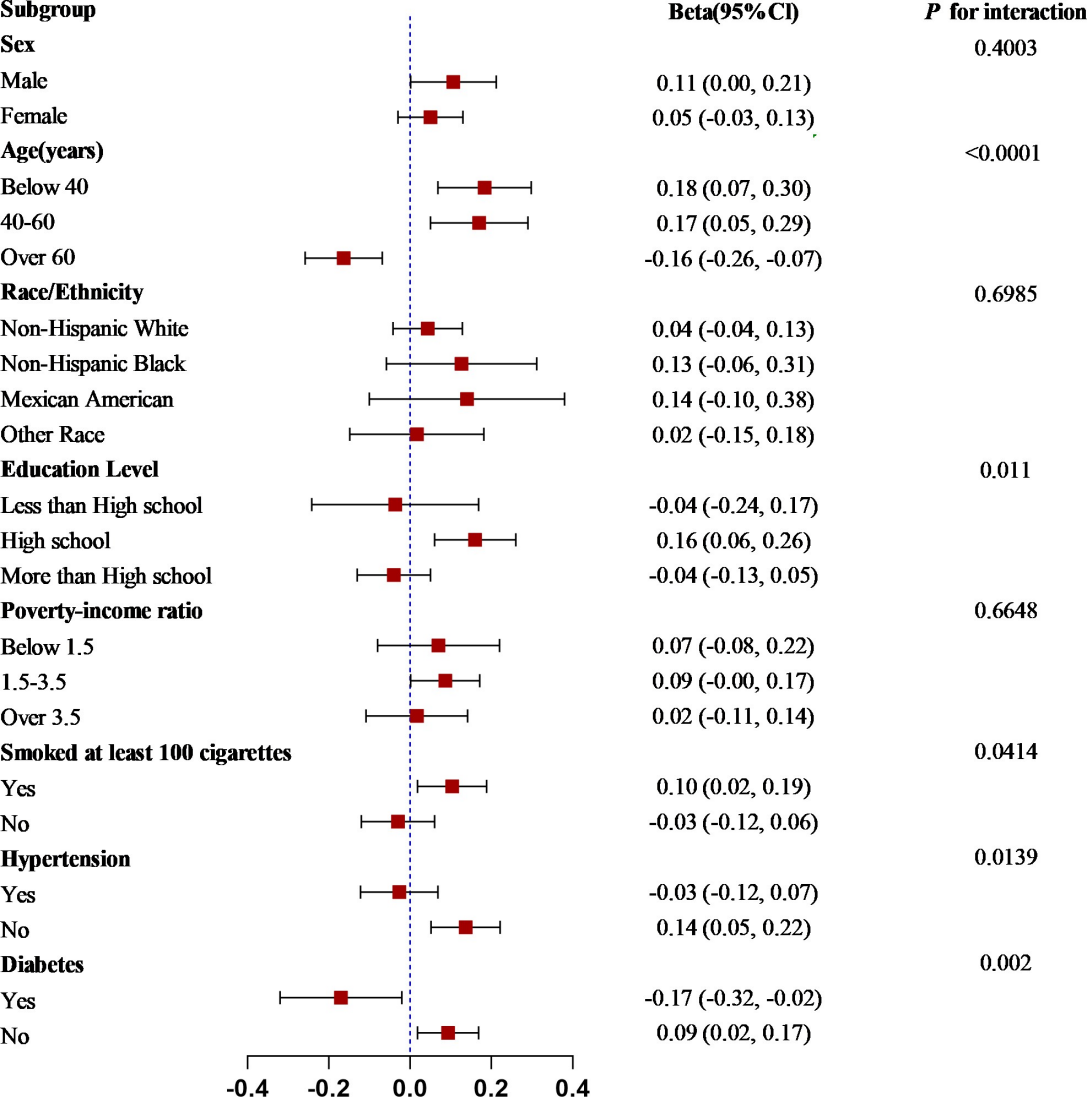
Subgroup analysis of the association between SII/100 and BMI.

Subgroup analyses and interaction tests were performed, stratified by sex, age, race, education level, poverty-to-income ratio, smoked at least 100 cigarettes, hypertension, and diabetes, to determine whether the association between the SII and BMI held for the broader community and to pinpoint any possibly distinct population contexts. Our results suggest that these associations are inconsistent, as shown in Fig 3.

A significant interaction was observed for the associations between the SII and BMI among subgroups characterized by age, education level, smoked at least 100 cigarettes, diabetes, and hypertension (all *p* < 0.05), whereas the associations between the SII and BMI were not statistically significant among subgroups characterized by sex, race, or poverty-to-income ratio. A significant positive correlation was observed between the SII and BMI in individuals aged < 60 years without comorbid hypertension and diabetes.

## Discussion

In this cross-sectional investigation, encompassing 4395 participants representative of the United States population, we observed a positive correlation between the SII and BMI. Further analysis revealed that the association between the SII and BMI was more apparent among individuals aged < 60 years without hypertension and diabetes. The SII showed an inverted U-shaped relationship with obesity, with the inflection point at 729.3. An SII < 729.3 was identified as an independent risk factor for obesity.

Very few studies have independently examined the association between the SII and BMI. Existing data indicate that inflammatory cytokines play an integral role in the mechanisms underlying obesity, and that sustained low levels of systemic inflammation accelerate the development of obesity [30]. In 1993, Hotamisligil et al. demonstrated for the first time that adipose tissue in individuals with obesity and animal models of obesity secreted elevated levels of TNF-α, and therefore revealed the close association between obesity and inflammation. Suppressing this release of pro-inflammatory factors from adipose tissue and promoting the secretion of anti-inflammatory factors alleviated chronic inflammation-induced tissue damage and impeded the development of various obesity-induced chronic diseases [17]. Skuratovskaia et al. studied peripheral blood and adipose tissue from 142 obese and 34 normal-weight individuals and showed that obese individuals had higher levels of CRP and TNF-α than those in normal-weight individuals [31]. In a study of adults aged 30–55 years by Kunz et al., individuals with a BMI > 30 kg/m^2^ were shown to have higher serum levels of CRP, IL-6, and TNF-α; there was a significant positive correlation between BMI and these indices [32]. Similar to previous findings, the current study identified a positive correlation between the SII and BMI. This implies that individuals with elevated levels of systemic inflammation may be more susceptible to obesity. In addition, this association was more pronounced in adults aged < 60 years without hypertension and diabetes. Younger adults have an active immune system that is reflective of the inflammatory state; however, with age, immune function declines, and inflammatory responses diminish [33]. Patients with hypertension and diabetes typically exhibit higher levels of inflammatory markers [34,35], which may mask the association between SII and obesity. Our results also showed a significant inverted U-shaped nonlinear relationship between the SII and BMI. Below the inflection point, the SII was significantly and positively correlated with BMI, probably reflecting increased levels of inflammatory mediators in the early stages of obesity, which affect insulin sensitivity and fat metabolism and promote fat accumulation. Above the inflection point, the SII was significantly and negatively correlated with BMI, possibly due to metabolic disorders triggered by severe inflammation or associated with chronic inflammatory diseases that lead to weight loss. The relationship between the SII and BMI has important clinical implications. First, the SII can be used as a simple but effective indicator of the inflammatory status of obese individuals, which can help in the early identification of obese individuals with a high inflammatory risk. Second, monitoring the SII, particularly in individuals aged < 60 years without hypertension and diabetes, may aid in the development of individualized prevention and intervention strategies to reduce the risk of obesity-related metabolic complications.

The possible mechanisms behind this positive correlation between the SII and BMI remain to be fully elucidated. In one potential mechanism, monocyte adhesion and transit and platelet engagement with neutrophils and lymphocytes during the inflammatory response leads to the secretion of numerous inflammatory factors, including TNF-a, IL-6, and IL-1 [36]. TNF-α, a crucial contributor to insulin resistance, suppresses the activity of insulin receptor tyrosine kinase and causes the phosphorylation of insulin receptor substrate 1, weakening insulin signaling, which in turn triggers metabolic disorders associated with obesity [37,38]. In a second possible mechanism, obesity leads to early and continuous infiltration of adipose tissue by neutrophils [39–41]. Neutrophils secrete inflammatory molecules, including myeloperoxidase, elastase, and IL-1β, and interact with adipocytes that recruit monocytes/macrophages [42]. In addition, obesity and the associated inflammation drive functional and phenotypic changes in T-cell populations, with elevations in CD4^+^ and CD8^+^ T-cell populations observed in obese individuals and in a mouse model of obesity [43]. In adipose tissue, there is a positive correlation between regulatory T cells and insulin sensitivity. However, the number of regulatory T cells decreases as obesity progresses [44,45]. Nevertheless, additional longitudinal investigations are required to definitively establish a causal association between the SII and obesity.

This study has several strengths. The sample was representative, and large enough to allow subgroup analysis. We adjusted for confounders to overcome the bias due to oversampling and produce robust results. Finally, we demonstrated the nonlinear association between the SII and BMI and the inflection points of this relationship. However, this study has certain limitations. This cross-sectional nature of the study meant that we could not determine a clear causal relationship between the SII and the risk of obesity. Moreover, even with appropriate adjustment for covariates, we were unable to completely rule out the impact of residual factors (such as inflammatory and autoimmune illnesses) on the outcomes. Our results suggest a positive correlation between the SII and BMI. However, further prospective studies are required to confirm a causal association.
